# Rapid diagnosis of experimental meningitis by bacterial heat production in cerebrospinal fluid

**DOI:** 10.1186/1471-2334-7-116

**Published:** 2007-10-10

**Authors:** Andrej Trampuz, Andrea Steinhuber, Matthias Wittwer, Stephen L Leib

**Affiliations:** 1 Division of Infectious Diseases & Hospital Epidemiology, Department of Internal Medicine, University Hospital Basel, Basel, Switzerland; 2 Infectious Diseases Research Laboratory, Department of Biomedicine, University Hospital Basel, Basel, Switzerland; 3 Institute for Infectious Diseases, University of Bern, Bern, Switzerland

## Abstract

**Background:**

Calorimetry is a nonspecific technique which allows direct measurement of heat generated by biological processes in the living cell. We evaluated the potential of calorimetry for rapid detection of bacterial growth in cerebrospinal fluid (CSF) in a rat model of bacterial meningitis.

**Methods:**

Infant rats were infected on postnatal day 11 by direct intracisternal injection with either *Streptococcus pneumoniae, Neisseria meningitidis *or *Listeria monocytogenes*. Control animals were injected with sterile saline or heat-inactivated *S. pneumoniae*. CSF was obtained at 18 hours after infection for quantitative cultures and heat flow measurement. For calorimetry, 10 μl and 1 μl CSF were inoculated in calorimetry ampoules containing 3 ml trypticase soy broth (TSB).

**Results:**

The mean bacterial titer (± SD) in CSF was 1.5 ± 0.6 × 10^8 ^for *S. pneumoniae*, 1.3 ± 0.3 × 10^6 ^for *N. meningitidis *and 3.5 ± 2.2 × 10^4 ^for *L. monocytogenes*. Calorimetric detection time was defined as the time until heat flow signal exceeded 10 μW. Heat signal was detected in 10-μl CSF samples from all infected animals with a mean (± SD) detection time of 1.5 ± 0.2 hours for *S. pneumoniae*, 3.9 ± 0.7 hours for *N. meningitidis *and 9.1 ± 0.5 hours for *L. monocytogenes*. CSF samples from non-infected animals generated no increasing heat flow (<10 μW). The total heat was the highest in *S. pneumoniae *ranging from 6.7 to 7.5 Joules, followed by *L. monocytogenes *(5.6 to 6.1 Joules) and *N. meningitidis *(3.5 to 4.4 Joules). The lowest detectable bacterial titer by calorimetry was 2 cfu for *S. pneumoniae*, 4 cfu for *N. meningitidis *and 7 cfu for *L. monocytogenes*.

**Conclusion:**

By means of calorimetry, detection times of <4 hours for *S. pneumoniae *and *N. meningitidis *and <10 hours for *Listeria monocytogenes *using as little as 10 μl CSF were achieved. Calorimetry is a new diagnostic method allowing rapid and accurate diagnosis of bacterial meningitis from a small volume of CSF.

## Background

Bacterial meningitis requires rapid diagnosis since a delay in antimicrobial therapy results in poor outcome [1,2]. Examination of CSF is of paramount importance for the diagnosis of meningitis. Accordingly, once contraindications have been ruled out, a lumbar puncture is performed in patients with suspected meningitis. Immediate examination of the CSF provides valuable information. A Gram stain of CSF indicates the presence of white blood cells, their approximate differential count and whether bacteria are present. However, CSF leukocyte counts may be normal or only marginally elevated in 5 to 10% of patients, especially in early stages of the infection [3]. Gram's staining and bacterial antigen tests of the CSF permit a rapid diagnosis, but have limited sensitivity [4].

Culture of CSF for the detection of bacterial growth is the gold standard for the diagnosis of bacterial meningitis [1]. Bacterial culture has a high sensitivity but results are typically available not earlier than on the following day. Molecular methods, such as polymerase chain reaction, are increasingly used in diagnostic microbiology, but are inherently restricted to detect only the pathogens specified by the test. Therefore, a rapid and sensitive tool to detect the presence of bacteria in CSF is needed to expedite diagnosis and to improve the management of patients with meningitis. *Streptococcus pneumoniae*, *Neisseria meningitidis *and *Listeria monocytogenes* are the main causative organisms responsible for over 80% of all cases of bacterial meningitis in adults [5].

Calorimetry is a nonspecific technique which allows direct measurement of heat generated by biological processes in the living cell. Microorganisms are reported to produce on average 1–3 pW heat per cell [6]. The exponential replication of microorganisms results in thermal changes over time that can be documented in real-time (i.e. heat power-time curve) by calorimetry. The current calorimeter can detect heat differences of less than 1 µW, which occur within minutes to hours if microorganisms grow exponentially in an appropriate medium. The detection time depends on the initial number of organisms, their replication rate and heat production per cell.

The present study was performed to demonstrate the proof of principle of calorimetry for the detection of bacterial growth in clinical samples. Here we used an infant rat model of bacterial meningitis due to infection with *Streptococcus pneumoniae*, *Neisseria meningitidis *and *Listeria monocytogenes *to evaluate the potential of isothermal calorimetry for rapid and sensitive detection of bacteria in small samples of CSF (i.e. 1 and 10 μL).

## Methods

### Model of meningitis

An established infant model of bacterial meningitis was used as described previously [7,8]. The animal studies were approved by the Animal Care and Experimentation Committee of the Canton of Bern, Switzerland, and followed National Institutes of Health guidelines for the performance of animal experiments. Briefly, nursing Wistar rats with their dams were purchased (Charles River, Germany). For each test organism, four animals (weighing 23.6 ± 1.1 g) were infected on postnatal day 11 by direct intracisternal injection of 10 μl saline containing 1 × 10^6 ^cfu/ml *S. pneumoniae*, 3 × 10^8 ^cfu/ml *N. meningitidis *and 2 × 10^5 ^cfu/ml *L. monocytogenes *using a 32-gauge needle. The pathogens investigated in the study show significant differences in virulence and pathogenicity. Therefore the number of bacteria used for infection needed to be adapted to generate reproducible infection and associated disease severity. Thus, for each pathogen the inoculum (cfu/ml) used for intracisternal infection was based on previously established disease models [9,10]. Control animals were injected with 10 μl sterile saline (n = 2) or sterile saline containing heat-inactivated (80°C for 20 min) *S. pneumoniae *at a concentration corresponding to 2 × 10^8 ^cfu/ml (n = 2).

### Infecting organisms

Clinical isolates of *S. pneumoniae *(serogroup 3), *N. meningitidis *(type C) and *L. monocytogenes *(serotype 4b) were used. The infecting organisms were previously passaged through infant rats in the course of experimental studies [9]. Organisms were grown on 5% sheep blood agar plates (Becton Dickinson, Heidelberg, Germany), cultured overnight in 10 ml of trypticase soy broth (TSB) for *S. pneumoniae *and *N. meningitidis*, or brain heart infusion (BHI) for *L. monocytogenes*, diluted in fresh medium, and grown to logarithmic phase at 37°C in 5% CO_2_. The culture broth was centrifuged for 10 minutes at 5,000 × g and the pellet was resuspended in sterile saline to the desired density for intracisternal injection. The accuracy of the inoculum dose was confirmed by quantitative cultures by serial dilution for each experiment. In addition, 10-fold of serial dilutions of test organisms were prepared in concentrations from 10^6 ^to 10^-1 ^cfu/ml as quantitative standards for comparison with CSF samples.

### CSF sampling and processing

At 18 h after infection, approx. 15 - 20 μl CSF was obtained by puncture of the cisterna magna and used for quantitative cultures and calorimetry. For quantitative culture, CSF was plated in 10-fold dilutions on 5% sheep blood agar plates (Becton Dickinson). For calorimetry, 10 μl and 1 μl CSF were added to 90 μl and 99 μl saline, respectively (total volume of 100 μl), and inoculated in calorimetry ampoules containing 3 ml TSB. Culture broth with 100 μl saline was used as control. Animals were sacrificed after CSF sampling with an overdose of pentobarbital (100 mg/kg intraperitoneally).

### Calorimetric equipment and measurements

An isothermal calorimetry instrument (Thermal Activity Monitor, Model 3102 TAM III, TA Instruments, New Castle, DE, USA) equipped with 48 channels was used to measure the heat flow at 37°C precisely controlled within 0.0001°C. The calorimetric sensitivity according to the manufacturer is ± 0.2 μW. Heat generated or absorbed was continuously measured in air-tightly sealed 4-ml glass calorimetry ampoules, containing the test or control samples; the gas phase consisted of ambient air. Ampoules were sequentially introduced into the calorimetry instrument and remained 15 minutes in the thermal equilibration position before lowering into the measurement position. Heat flow was measured for up to 48 hours at 10 s intervals. After the measurement was completed, the content of each calorimetry ampoule was cultured to confirm the inoculated microorganisms in infected animals or sterility of the negative controls.

### Analysis of calorimetric data

Calorimetric detection time was defined as the time from insertion of the ampoule into the calorimeter until the growing culture produced an exponentially rising heat flow signal exceeding 10 μW. Peak heat flow was defined as the highest value of the heat power-time curve. Total heat was determined by integration of the area below the heat flow-time curve. Data analysis was accomplished using the manufacturer's software (TAM Assistant, TA Instruments, New Castle, DE, USA) and Origin 7.5 (Microcal, Northampton, MA, USA).

## Results

### Bacterial meningitis in infant rats

At 18 hours after intracisternal injection, all infected rats suffered from meningitis as evidenced by lethargy, diminished weight gain and positive CSF cultures. Table 1 shows the bacterial density in CSF at 18 hours after infection. In control animals, injected with sterile saline or heat-inactivated bacteria, no signs of meningitis were observed at 18 h after infection and no bacteria grew in CSF cultures.

**Table 1:** Bacterial concentration and calorimetric detection time of 1 μl and 10 μl CSF punctured 18 h after intracisternal injection of bacteria (infected cases) or sterile fluid (controls). Calorimetric detection time is defined by the time from insertion of the ampoule into the calorimeter until the heat flow exceeded 10 μW. Data represent mean values ± SD.

**Intracisternal injection (No. of animals)**	**Bacterial titer in CSF (cfu/ml)**	**Calorimetric detection time (h)**^a^		**Peak heat flow (μW)**^b^
			
		**10 μl CSF**	**1 μl CSF**	
*S. pneumoniae *(n = 4)	1.5 ± 0.6 × 10^8^	1.5 ± 0.2	2.8 ± 0.3	342 ± 29
*N. meningitidis *(n = 4)	1.3 ± 0.3 × 10^6^	3.9 ± 0.7	5.5 ± 1.1	75 ± 24
*L. monocytogenes *(n = 4)	3.5 ± 2.2 × 10^4^	9.1 ± 0.5	13.0 ± 1.0	340 ± 15
Heat-inactivated *S. pneumoniae *(n = 2)	No growth	Negative	Negative	<10
Saline control (n = 2)	No growth	Negative	Negative	<10

NOTE. CSF, cerebrospinal fluid.

^a ^Defined as the time from the start of measurement until the detected heat flow exceeds 10 μW.

^b ^Peak heat flow was the highest value of the heat power-time curve.

### Heat power-time curves from CSF and standard bacterial dilutions

**Figure 1:**
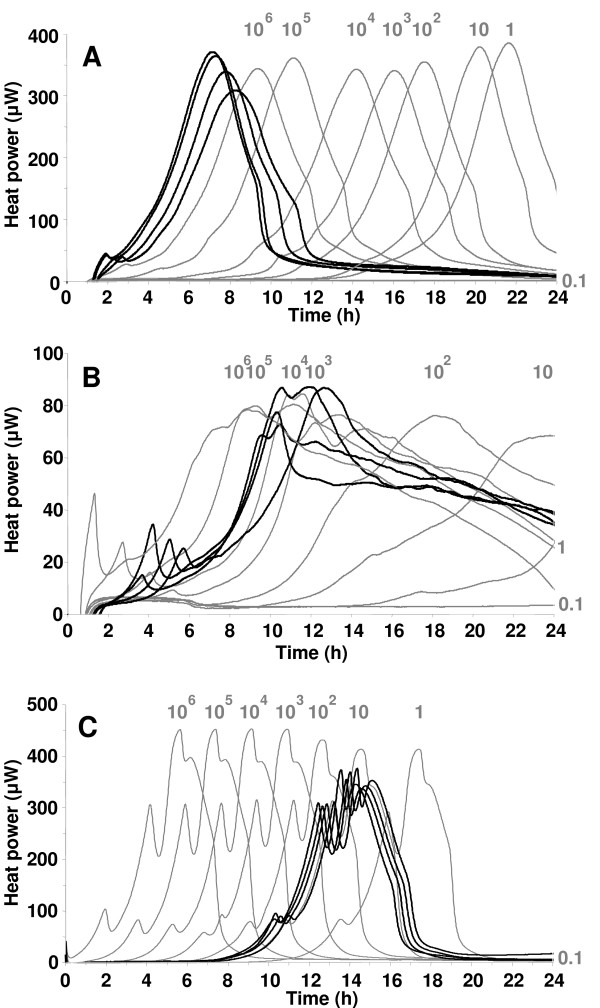
Heat power-time curves for *S. pneumoniae *(A), *N. meningitidis *(B) and *L. monocytogenes *(C). Black lines represent heat produced from 10-μl CSF samples drawn at 18 hours after infection from four rats per group and bacterial species. The corresponding mean (± SD) bacterial titer were 1.5 ± 0.6 × 10^8 ^cfu/ml for *S. pneumoniae*, 1.3 ± 0.3 × 10^6 ^cfu/ml for *N. meningitidis *and 3.5 ± 2.2 × 10^4 ^cfu/ml for *L. monocytogenes*. CSF from non-infected rats (controls) produced no heat. Grey lines represent serial 10-fold bacterial dilutions from 10^6 ^to 10^-1 ^cfu/ml.

Heat signal was detected in CSF samples from all infected animals, whereas those from non-infected animals generated no detectable heat (<10 μW). Figure 1 shows heat power-time curves from 10-μl CSF samples (bold lines) in correlation with heat produced by serial 10-fold bacterial dilutions ranging from 10^6 ^to 10^-1 ^cfu/ml (grey lines). The heat curves of CSF from different individual animals infected by the same organism showed similar characteristics. For example, *S. pneumoniae *showed a discrete initial peak heat flow at 40 μW, followed by a second peak at about 350 μW. *N. meningitidis *demonstrated a clear initial peak, followed by a higher peak at approximately 80 μW. Replicates of *L. monocytogenes *showed three peaks, at ≈ 80 μW, ≈ 300 μW and ≈ 350 μW. The shape of the curves was dependent on the growth medium in the calorimetry ampoule, but independent from the initial bacterial concentration (data not shown). The bacterial titer in CSF at 18 hours after infection correlated with the standard dilutions for *S. pneumoniae*, but was two orders of magnitude lower for *N. meningitidis *and three orders of magnitude lower for *L. monocytogenes*. The total heat was the highest in *S. pneumoniae *ranging from 6.7 to 7.5 Joules, followed by *L. monocytogenes *(5.6 to 6.1 Joules) and *N. meningitidis *(3.5 to 4.4 Joules). CSF from animals that had been injected with heat-killed bacteria resulted in power-time curves very similar to those of the medium-controls. The values always remained below the 10 μW positivity limit and continuously decreased towards baseline.

### Calorimetric detection time from CSF

Table 1 summarizes the calorimetric detection times for the different pathogens generated from 10 μl and 1 μl of CSF. The detection time is defined by the time elapsed from the start of measurement until the detected heat flow exceeds 10 μW. In 10-μl CSF samples, *S. pneumoniae *was detected after a mean ± SD of 1.5 ± 0.2 hours, followed by N. meningitidis after 3.9 ± 0.7 hours and by L. monocytogenes after 9.1 ± 0.5 hours.

### Calorimetric detection limit

**Figure 2:**
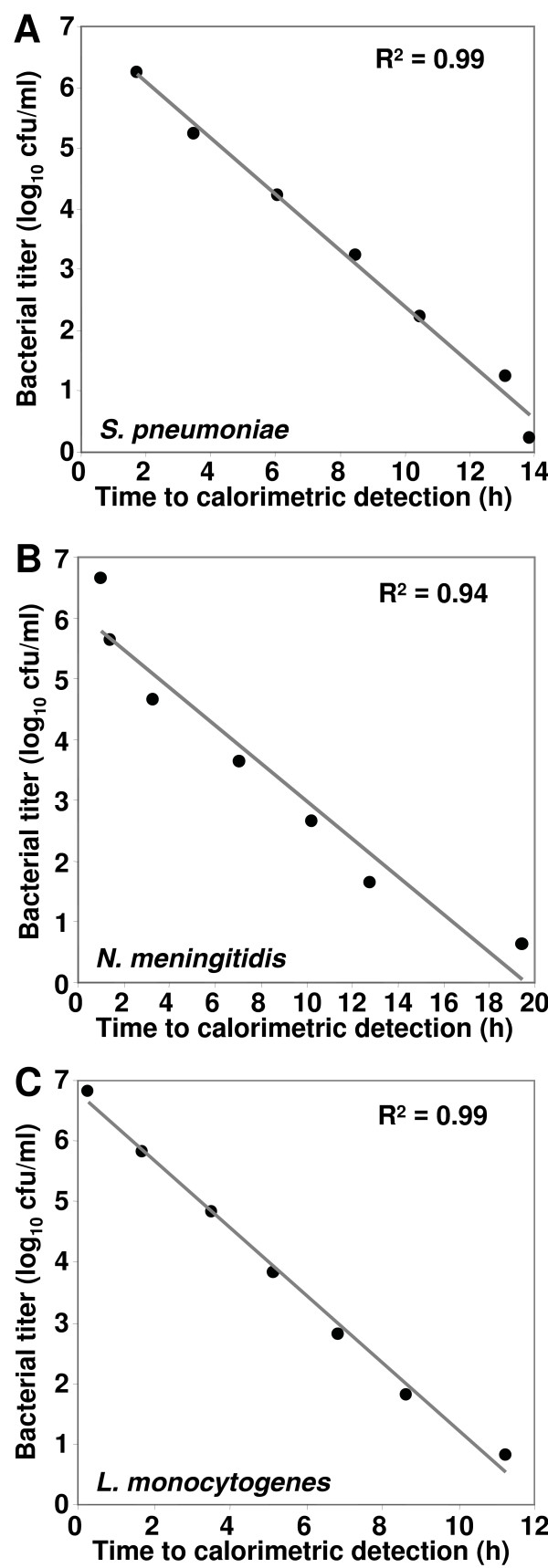
Correlation between the time to calorimetric detection and initial bacterial titer based on serial 10-fold dilutions ranging from 10^6 ^to 10^-1 ^cfu/ml of (A) *S. pneumoniae*, (B) *N. meningitidis*, and (C) *L. monocytogenes*. R^2 ^denotes the coefficient of determination using simple linear regression.

As determined from standard bacterial dilutions by linear regression analysis, the lowest detectable bacterial titer by calorimetry was 2 cfu for *S. pneumoniae*, 4 cfu for *N. meningitidis *and 7 cfu for *L. monocytogenes *using TSB (Figure 2). The lowest bacterial titer was detected after 15.2, 19.7 and 12.2 hours of incubation, respectively.

### Influence of media on calorimetric detection time

In addition to TSB, BHI was used as an alternative growth medium for the detection of *S. pneumoniae *and *L. monocytogenes*. CSF drawn from animals was split into identical volumes of 10 μl and inoculated in parallel in calorimetry ampoules containing TSB or BHI. The mean detection time (± SD) in TSB was 1.71 ± 0.37 hours shorter for *S. pneumoniae *and 1.17 ± 0.87 hours shorter for *L. monocytogenes* compared to BHI. Similarly, the peak heat flow of CSF in TSB was 174 μW higher for *S. pneumoniae *and 69 μW higher for *L. monocytogenes *as compared to BHI.

## Discussion

Bacterial meningitis is a medical emergency situation associated with a substantial morbidity and mortality. Despite appropriate antibiotic therapy recent studies document a mortality rate of up to 30% and the occurrence of permanent sequelae due to neuronal injury in up to 50% of the survivors [1,2,11]. A number of studies suggest an association between adverse clinical outcome of bacterial meningitis and the time until initiation of antibiotic therapy after presentation [1,2,12,13]. Thus, rapid diagnosis and start of antibiotic therapy should be a major therapeutic goal for physicians treating patients with bacterial meningitis [1]. The ongoing search for alternative methods to diagnose bacterial meningitis more rapidly, such as polymerase chain reaction based detection of bacterial DNA and RNA or latex agglutination tests in CSF, and the variety of published clinical prediction rules and algorithms of clinical and CSF parameters are testimony to the fact that the available diagnostic tools are still unsatisfactory in the clinical situation [14].

Calorimetry has been widely used in biology, chemistry, pharmacology, biotechnology and ecology because of its high sensitivity, accuracy and simplicity [15]. However, its clinical application was previously hindered by insufficiently sensitive equipment, low throughput capacity and the lack of appropriate software. In past years, the technology improved and highly sensitive instruments are now available, capable to detect temperature differences of <10^-6^°C [16]. The exponential increase of the heat signal reflects the exponential increase in bacterial numbers during the logarithmic growth phase [17]. In a recent study the potential of calorimetry to detect contaminated platelets concentrates was evaluated [18]. The present report is the first about the application of calorimetry as diagnostic tool in clinical samples, e.g. CSF in bacterial meningitis.

The detection principle relies on the heat generation of viable, replicating bacteria from CSF in culture medium that is continuously measured by isothermal calorimetry. The sensitivity of calorimetry compared to standard culture methods is equivalent when identical volumes of sample fluid are used in both methods, as the prerequisite for both detection methods is bacterial multiplication in culture. However, due to the more sensitive and continuous measurement principle of calorimetry (i.e. monitoring of heat production), the detection time is significantly shorter than with conventional cultures, which is based on detection of growth on agar plates or in broth.

All infected animals in the present study were diagnosed by calorimetric analysis of punctured CSF samples within hours and with a high discriminative power compared to sterile inflammation or medium controls. The present data are in agreement with the recent finding that the mode of growth observed in bacteria during invasive tissue infection, e.g. growth in cerebrospinal fluid, differs significantly from bacterial growth in liquid medium [19]. The metabolic activity of inflammatory cells in CSF, mainly leukocytes, did not interfere with the increasing heat signal of the multiplying bacteria. For all tested bacterial species the detection time, corresponding to the time until a threshold level of 10 μW was reached, was dependent on the concentration of bacteria. Initial numbers of less than 10 cfu of organisms per calorimetric ampoule could be detected.

## Conclusion

In the present approach, detection times of <4 hours for *S. pneumoniae *and *N. meningitidis *and <10 hours for *Listeria monocytogenes *using as little as 10 μl CSF were achieved. In the clinical situation larger volumes of CSF are usually available from patients, which may further shorten the detection time. This method is particularly well suited in situations where CSF volumes are limited (e.g. in newborns and infants). Calorimetric detection of bacterial growth may thus provide an accurate tool to rapidly diagnose bacterial meningitis with the benefit of low labor intensiveness, few handling steps, potential of complete automation, and minimal contamination and biohazard risk due to the closed ampoule system.

In addition, we found that TSB is an appropriate medium for rapid detection of all tested microorganisms in this study. The differences in the power-time curves of *L. monocytogenes *after (CSF) or without (standard curves) passages through the animal could be due to adaptation of the pathogen that may be associated with a prolonged lag phases and longer generation times.

This study demonstrates that calorimetry is a promising novel approach for rapid and accurate diagnosis of bacterial meningitis, allowing detection of multiplying bacteria in small volumes of CSF (1–10 μl) within hours.

## Abbreviations

Cerebrospinal fluid – CSF

Trypticase soy broth – TSB

Brain heart infusion – BHI

Standard deviation – SD

## Competing interests

The author(s) declare that they have no competing interests.

## Authors' contributions

All authors read and approved the final manuscript. AT, AS and SLL contributed equally for study design, coordination, interpretation of results and writing of the manuscript. AS, MW and SLL participated in data collection and experimental procedures.

## Pre-publication history

The pre-publication history for this paper can be accessed here:


